# Improving Dietary Zinc Bioavailability Using New Food Fortification Approaches: A Promising Tool to Boost Immunity in the Light of COVID-19

**DOI:** 10.3390/biology12040514

**Published:** 2023-03-29

**Authors:** Marouane Chemek, Ammar Kadi, Svetlana Merenkova, Irina Potoroko, Imed Messaoudi

**Affiliations:** 1 Department of Food and Biotechnology, South Ural State University, 454080 Chelyabinsk, Russia; 2 Laboratoire LR11ES41 Génétique Biodiversité et Valorisation des Bio-Ressourcés, Institut Supérieur de Biotechnologie de Monastir, Universitéde Monastir, Monastir 5000, Tunisia

**Keywords:** zinc, bioavailability, immunity, COVID-19, food

## Abstract

**Simple Summary:**

Zinc is a powerful immunomodulatory trace element, and its deficiency in the body is closely associated with changes in immune functions and viral infections. Control of the amount of this element in the diet is especially important for populations at risk of zinc deficiency. Using convergent approaches such as micro- and nano-encapsulation, scientists have recently come up with new ways to treat zinc deficiency and make zinc more bioavailable.

**Abstract:**

Zinc is a powerful immunomodulatory trace element, and its deficiency in the body is closely associated with changes in immune functions and viral infections, including SARS-CoV-2, the virus responsible for COVID-19. The creation of new forms of zinc delivery to target cells can make it possible to obtain smart chains of food ingredients. Recent evidence supports the idea that the optimal intake of zinc or bioactive compounds in appropriate supplements should be considered as part of a strategy to generate an immune response in the human body. Therefore, controlling the amount of this element in the diet is especially important for populations at risk of zinc deficiency, who are more susceptible to the severe progression of viral infection and disease, such as COVID-19. Convergent approaches such as micro- and nano-encapsulation develop new ways to treat zinc deficiency and make zinc more bioavailable.

## 1. Introduction

Food science has just begun to scratch the surface of the potential contribution that food may make to health and well-being. We anticipate that the pandemic health emergency and the urgent need to strengthen prevention and control strategies will contribute to the mobilization of research efforts on innovative topics including nutraceutical activities, food/drug interactions, improved bioavailability, and novel formulations of food components. When compared with traditional pharmacological methods, food-based techniques often have the benefit of producing fewer unwanted side effects [1]. The fortification of natural molecules in food in order to provide the proper intake of macro- and micronutrients and to help reduce the severity of infections is considered as one of the current and promising food-based approaches in the prevention and treatment of disease and viral infections, including coronavirus disease 2019 (COVID-19) [1]. One of the natural elements present on earth and available in human food is zinc (Zn). The structural, catalytic, and regulatory roles that Zn plays in the body make it indispensable for a wide variety of physiological processes, such as those involved in development, metabolism, immunological function, and brain function [2,3]. A significant portion of the worldwide burden of malnutrition may be caused by Zn deficiency, as it is ubiquitous among communities all over the world [4]. Researchers estimate that Zn depletion in the diet of the world’s population ranges from 17% to 20%, mostly in Asian and African countries [4]. Zn deficiency is most common in the elderly, vegetarians, alcoholics, pregnant and lactating women, and people with chronic diseases [5]. Zn deficiency occurs for several reasons: lack of the element in the diet, insufficient Zn absorption, and increased Zn loss from the body [6,7]. This leads to a number of functional disorders of hormones, the central nervous system, and reduced immunological protection [6,7]. Excess Zn also leads to acute effects and chronic diseases. To prevent Zn deficiency, it is necessary to improve Zn bioavailability in order to reach the recommended optimum Zn intake of 11 mg/day for males and 8 mg/day for women [8]. In this regard, a few different approaches exist including a varied diet, supplementing food, and the fortification of the diet. However, these are not cost-effective and poorer populations are unable to afford them. Furthermore, minerals, such as Zn, can be exposed to oxidation and interact with food macromolecules, such as phytate that reduces the bioavailability of Zn [9]. Therefore, successful Zn fortification strategies should consider its bioavailability in the whole diet.

The purpose of this review article is to provide a synopsis of the many pharmacological and physiological roles of Zn and its immunomodulatory qualities, both of which have the potential to be useful in the treatment and prevention of viral infections, such as COVID-19. It also aims to outline the strategies that have been used to boost the bioavailability of Zn in food fortification procedures.

## 2. Overview of the Biological and Physiological Functions of Zinc

Zn is recognized as a physiological metal that is essential to the metabolism of most living beings. Since there is no specialized zinc storage system in the human body, a sufficient daily intake of Zn is required to achieve optimum physiological function [5]. Zn is involved in the main metabolic pathways, either as a cofactor or as a constituent of the structure of enzymes [6]. More than 2000 finger proteins, including transcription factors, steroid receptors, hormones, and enzymes, have been shown to rely on Zn for the maintenance of their three-dimensional structures [10]. In light of these facts, Zn is involved in a broad variety of biological processes, such as cell proliferation and differentiation, the cell cycle, the homeostasis of reactive oxygen species (ROS), and the immunological response [6,10]. Variations in normal Zn concentrations may lead to several fatal illnesses, alterations in immune responses, stunted development, and neurological problems, which have all been linked to Zn deficiency [11] in addition to increasing susceptibility to environmental pollutant toxicity [12,13]. Zn supplementation prevents these effects [14,15]. Therefore, maintaining Zn homeostasis is essential for the well-being of an organism.

## 3. Zinc Homeostasis and Bioavailability

### 3.1. Zinc Homeostasis in the Body

**Figure 1:**
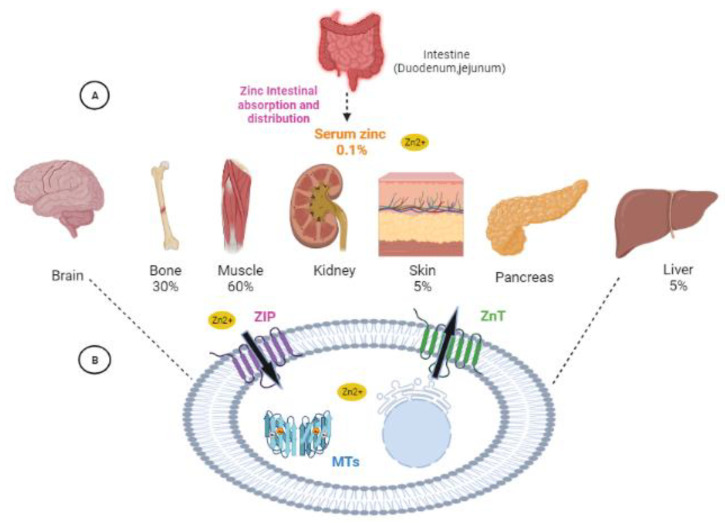
Zn homeostasis in the body and cellular mechanism. (**A**) Zn distribution in the body. (**B**) Cellular mechanism of Zn homeostasis: cellular Zn homeostasis is controlled by the cooperative function of MTs and two Zn transporter families (ZIP and ZnT). The arrows indicate the transmembrane pathways of Zn using ZIP and ZnT transporters.

About *2* g of Zn may be found in an adult human body [16]. Zn is distributed in the body as follows: 60% is in skeletal muscle, 30% in bone, 5% in the liver and skin, and the other 5% is elsewhere [17] (Figure 1A). Only 0.1% of the Zn in the body is found in serum, and of that, 80% is loosely attached to albumin and 20% is securely bound to 2-macroglobulin [18]. The human body replenishes 0.1% of its Zn needs every day via food. Duodenal and jejunal Zn absorption is tightly controlled, increasing by as much as 90% in the presence of a deficient diet, whereas Zn excretion in the presence of an overabundance is aided by gastrointestinal secretion, the shedding of mucosal cells and integument, and renal excretion [17].

### 3.2. Cellular Mechanism of Zinc Homeostasis

Proteins involved in Zn transport, storage, and release keep cellular Zn homeostasis in check (Figure 1B). Important functions in cellular Zn homeostasis are played by metallothioneins (MTs) and two Zn transporter families, Zrt- and Irt-like proteins (ZIP, solute carrier 39A [SLC39A]) and Zn transporters (ZnT, SLC30A) [19,20]. Whereas ZnT transporters serve as cation diffusion proteins involved in Zn efflux and compartmentalization, ZIP transporters are responsible for transporting Zn from the extracellular space or intracellular organelles into the cytosol [20]. MTs are ubiquitous low-molecular-weight proteins characterized by their richness in cysteine and Zn (6 to 7 atoms/protein molecule), as well as by a remarkably conserved structure during evolution. Under normal physiological conditions their primary function is to regulate the internal homeostasis of copper (Cu) and Zn by keeping the cytoplasmic concentration of essential metals low when they are free through the binding of excess metals in a non-toxic form [12].

### 3.3. Zinc Bioavailability

The term “bioavailability” refers to the degree to which an organism is able to absorb and use the nutrients it consumes. The bioavailability of minerals can be evaluated using the methods based on measuring mineral element accumulation in certain sensitive tissues. As previously discussed, high levels of dietary Zn may negatively affect the liver and pancreas [21]; therefore, the amount of Zn stored in the liver and pancreas is utilized to calculate the bioavailability of Zn from various Zn sources [22]. Some research suggests that plant intake contains Zn in the form of inorganic ions, whereas animal products contain Zn in the form of organic protein complexes [23]. However, no absorption experiments (i.e., employing isotopic labeling) have been undertaken to assess if these forms of Zn vary in bioavailability. The amount of dietary Zn normally causes an increase in Zn absorption that is greatly impacted by the general composition of the diet [24]. Zn absorption is decreased by the phytate used by plants to store phosphorus. In the lumen of the small intestine, phytate sequesters Zn from the active transport systems found on the cell surfaces by binding Zn ions with a high affinity [25]. Unlike phytate, which is reliant on the source of the protein, dietary protein itself improves Zn absorption. Foods made from animal sources are high in protein and free of phytate [26]. As a result, adding animal sources of protein without phytate interference enhances fractional Zn absorption from the diet. In contrast, the largest phytate level is often seen in plant sources of protein. Although increasing plant protein boosts Zn absorption, if phytate is not lowered this impact can be negated [25].

Serosal variables affect Zn absorption. Due to its role as a basolateral Zn acceptor, serum albumin facilitates Zn release from enterocytes into the bloodstream [9]. The liver may also play a significant role in secreting humoral factors controlling intestinal Zn absorption; systemic humoral factors, such as hepcidin, seem to regulate the ZnT-1-mediated export of Zn by intestinal cells [27], and Zn interacts with other food components resulting in the augmentation or inhibition of its absorption through the intestinal wall and into the bloodstream [9]. Therapeutic or experimental iron (Fe), calcium (Ca), and cadmium (Cd) supplementation has been shown to negatively affect plasma Zn levels, for example, in pregnant women [28] and during lactation [19]. Physiological states are an important factor influencing Zn absorption and bioavailability, along with dietary promoters such as animal protein and low-molecular-weight organic compounds and dietary inhibitors such as phytate, serosal factors, and possibly interaction with essential (Fe and Ca) or toxic (Cd) metals. The demands of the mother’s tissues, the developing fetus, and a nursing infant may require an increase in fractional Zn absorption in humans [29,30,31].

## 4. Zinc as an Immunomodulatory Element and Its Implications for COVID-19

### 4.1. Zinc and Immunity

**Figure 2:**
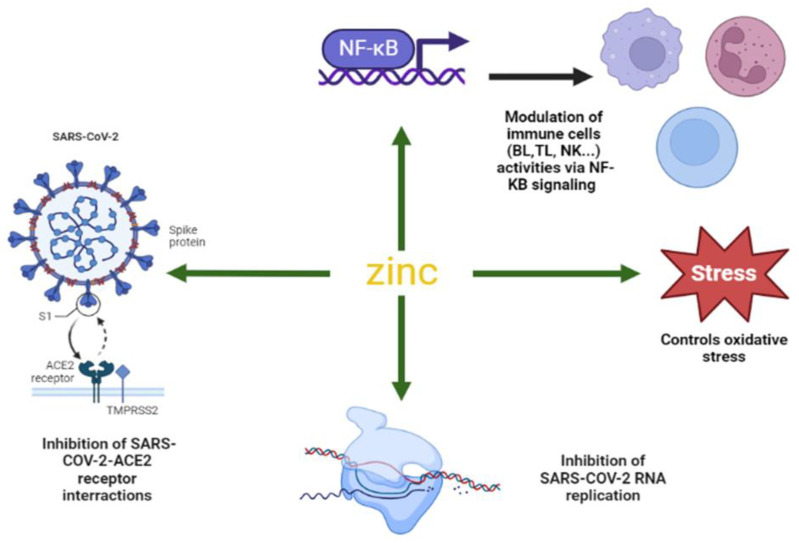
The molecular mechanisms of the anti-viral and immunomodulatory roles of zinc during COVID-19 infection. (ACE2): Angiotensin-converting enzyme 2, (BL): B lymphocytes, (TL): T lymphocytes, (Nk): Natural killer cells. [35,56,57].

Zn is an essential micronutrient involved in the regulation of innate and adaptive immune responses [32]. Many chronic illnesses (such as cirrhosis, renal insufficiency, malignancies, and auto-immune arthritis) that result in a Zn deficiency are linked to an increase in the risk of bacterial or viral infections. This reveals that Zn plays a crucial role in maintaining the homeostasis of a variety of immune cells and processes, including natural killer (NK) cells, T lymphocytes (TL), B lymphocytes (BL), cytokine synthesis, and other immunological components [32]. The maintenance of a healthy immune function relies on maintaining optimal Zn levels. Within this concept, Zn deficiency causes atrophy in the thymicolymphatic system, a decrease in macrophage and TL functions, the depression of cellular immunity, and a decrease in immunoglobulin levels. Zn deficiency also leads to the suppression of the activity of NK cells since Zn is one of the main cofactors of thymulin, a stimulator of natural killer cells [7,33,34]. Supplemental Zn corrects the negative impact of Zn homeostasis disruption or a Zn shortage on the immune system and on infections with microorganisms including bacteria, fungi, and viruses, especially respiratory viral infections [34,35]. The physiological mechanism by which Zn modulates the immune response is complicated and is still under investigation. However, the modulation of the expression of inflammatory cytokines, the control of oxidative stress, and the control of the transcription component nuclear factor kappa B (NF-B), all of which are essential for good immunological function, figure among the most described mechanisms [34] (Figure 2). Studies have confirmed the essential role of Zn for the human microbiome, as well as the influence of the Zn-induced modulation of the intestinal microflora and the accumulation of metabolites on the immunological regulation of the body’s status [36]. Zn impacts numerous parts of the immune system, from the skin’s barrier to gene regulation inside cells, and its molecular processes for modulating immunological response during infection have been investigated for decades, as reviewed by [6,35]. Recently, it has been documented that epigenetic mechanisms regulate the expression of various physiological-function-related genes including those of the immune system, modifying the development of the innate and adaptive immune responses [37]. Epigenetic regulation mainly includes DNA modifications, histone post-translational modifications (PTMs), and noncoding-RNA-associated silencing. Any alterations in epigenetic markers may result in the decline of immune function and this can be transferred through generations [38]. It has also been established that Zn is required for the activity of various epigenetic enzymes, such as DNA methyltransferases (DNMTs), histone acetyltransferases (HATs), histone deacetylases (HDACs), and histone demethylases, which possess several Zn binding sites [39]. Thus, the dysregulation of Zn homeostasis can lead to epigenetic alterations and to a disorder in the immune response, increasing the risk of viral infection. Interestingly, the transcriptional gene regulation of the binding to metal response elements (MRE) or the Zn transcriptional regulatory element (ZTRE), two Zn-dependent transcriptional activators/repressors involved in the gene expression of protein that regulate Zn homeostasis (ZIP, ZnT and MT), are controlled by epigenetic mechanisms such as DNA methylation [40].

From this evidence, it is clear that Zn plays an important role in keeping the immune system functioning and that Zn deficiency could increase the susceptibility to viral infection. Zn has been shown to exert direct inhibitory effects against a variety of viruses, including SARS-CoV-2 [35,41].

### 4.2. Zinc and COVID-19

There is mounting evidence that adequate Zn consumption or suitable supplementation can mitigate the impacts of COVID-19 [42,43]. Clinical studies for COVID-19 are now testing Zn therapies (alone and in combination with other medications) [44,45]. A recent pre-proof medical report based on four hospitalized patients clinically diagnosed with COVID-19 and treated orally with high doses of Zn salts (15–23 mg/day) showed significant improvement in the disease after one day of treatment, suggesting that high-dose Zn therapy may play a role in clinical recovery as an example of Zn treatment not as an adjuvant to other drugs [46]. Additionally, anti-SARS-CoV-2 medications such chloroquine (CQ) raise intracellular concentrations of this micronutrient [44]. Furthermore, several recent in silico studies have demonstrated by molecular docking analysis that Zn could have a strong affinity and inhibit the main cellular compound targets of SARS-CoV-2, including the angiotensin-converting enzyme 2 (ACE2) receptor (which could be considered the main entry target of the virus), RNA-dependent RNA polymerase (RdRp), and the main protease activities (3-chyomotrypsin-like protease (3CLpro), etc.) used for virus replication [47,48,49,50]. Interestingly, the results from recent in vitro studies have confirmed the findings from in silico screening and revealed that Zn alone or in conjugation with other compounds (Zn ionophore, drug, etc.) could inhibit the virus at several steps in its lifecycle (entry, replication, and assembly) by downregulating the expression and activities of the cellular machinery used by the virus [51,52,53,54] (Figure 2). In addition, the in vitro study by Ghareeb et al. (2021) suggests that Zn could be useful to cure a second bacterial infection that took place in hospitalized COVID-19 patients [55].

Due to the fact that Zn deficiency negatively affects immune system functions, controlling the amount of this element in the diet is especially important for populations at risk of zinc deficiency, such as infants and young children, pregnant women, and the elderly, who are all more susceptible to the severe progression of viral infection and diseases such as COVID-19. Possible interventions include food fortification. Studies evaluating the impact of Zn-fortified foods on the human immune system are scarce [58,59,60] and further research is needed in this field.

## 5. Zinc and Food Products

### 5.1. Zinc Content in Food Product

According to the Food and Agriculture Organization (FAO) and the World Health Organization (WHO), it is important to consider the state of Zn in the agrobiological chain (soil–water–plants) and the conditions affecting the bioavailability of Zn, as well as strategies to reduce Zn deficiency in human diets. In nature, Zn is accumulated in plant roots from the soil as organic compounds and is then transferred to shoots, leaves, and fruits [61].

Different levels of Zn content were observed in plant products related to the origin of products, cultivation methods, and genotype [62]. A high content of the Zn element was found in seeds and cereal kernels (up to 3400 µg/100 g) and in nuts (up to 2972 µg/100 g). The content of Zn in traditional foods varies widely. Thus, the highest concentrations of the element are found in products of animal origin [63]: in by-products (3630–4550 µg Zn/100 g), in meat content (1490–3600 µg Zn/100 g), and in milk content (310–445 µg Zn/100 g). Moreover, oysters are widely considered one of the richest food sources for Zn [64]. The Zn content in plant foods, cereals, and beans is comparable with that of meat and by-products. However, one should consider the low bioavailability of nutrients from plant materials due to the ability of phytic acid to bind metal ions [65].

Environmental pollution has a significant impact on the concentration of Zn in plant and animal foods. In cereal crops in urban areas, Zn concentration increases by 12–15%, and in areas adjacent to metal processing industries, the content of the element increases by 40–60% [8].

### 5.2. Fortification/Biofortification of Food with Zinc

Malnutrition caused by a lack of micronutrients has emerged as a significant threat to public health in recent decades and requires prompt treatment. The lack of access to sufficient amounts of nutritious food, an imbalanced diet, an absence of dietary variety, and other factors are major contributors to malnutrition. Zn deficiency in the soil leads to inadequate consumption of Zn by humans in their regular diet, which in turn decreases the amount of Zn that is bioavailable [66]. There are a few different approaches that may be taken to treat this illness, including the intake of a varied diet, supplementing food, and diet fortification. However, these are not cost-effective and poorer populations are unable to pay for them. In this context, agronomic biofortification using Zn fertilizers is a sustainable technique for reducing the micronutrient shortage in the population, particularly for the populations of low-income nations throughout the world [67]. The main aim of Zn biofortification is to stimulate the accumulation of the essential element in the edible part of the plant through the use of mineral fertilizers or the selection of plant varieties [67]. The Zn content in plants may be easily and cheaply increased using this approach. Genetic biofortification, though time consuming, is also an effective method for lowering the risk of micronutrient deficiencies [68]. On other hand, the technological processing of food raw materials results in a decrease in the content of nutrients, which leads to an insufficient intake of minerals and vitamins. To compensate for micronutrient deficiencies, various approaches towards food fortification are used, combining the use of functional food additives, which are sources of minerals, depending on their solubility, bioavailability, digestibility, and cost [69]. Therefore, the choice of a suitable food matrix for fortification is crucial. It is recommended to fortify products intended for mass or specific consumption, including cereals, fruits, and dairy-based products. Milk and dairy products are also candidates for Zn fortification because of their wide consumption in many countries, their high nutritional value, and their buffering effect on digestion and absorption. The dietary co-intake of dairy products can also increase Zn absorption from other food sources [70,71]. Another property that makes dairy products a logical choice for Zn fortification is their low pH level, which preserves the stability of trace elements [70].

In the same framework, many Zn precursors, such as Zn oxide (ZnO), Zn sulfate (ZnSO_4_), and Zn citrate (ZnCi), have been widely used as supplements and fortifier compounds in food products [71,72,73,74]. ZnO is the most frequently used fortifier due to its cheapness [72]. ZnSO_4_ contains approximately 23% of elemental Zn, which makes it a potential candidate for food fortification to combat Zn deficiency [72]. ZnCi is much less used in food fortification, but a recent clinical study has demonstrated that Zn absorption in young adults receiving ZnCi supplementation was much higher than in those receiving ZnO [75].

As a result, agronomic and genetic biofortification alongside the use of functional food additives can be extremely beneficial in reducing micronutrient deficiency. They also assist in enhancing the amount of various micronutrients that are present in the food basket.

## 6. Zinc Encapsulation and Chelation as Novel Approaches to Increase Zinc Bioavailability in Food Fortification/Biofortification

The main challenges in the fortification/biofortification of food with Zn are related to increasing its solubility and bioavailability, reducing the interaction of the trace element with the food matrix, and leveling the negative impact on the flavor and aroma of the final product. In this regard, many research groups are developing new structures and strategies that could improve Zn bioavailability. Microencapsulation, nanotechnologies, and the use of biological compounds extracted from plants as Zn chelators are among the most recent methods used to increase Zn bioavailability in the fortification/biofortification of food products.

### 6.1. Chelating Zinc with a Biological Compound

For over 70 years, biological chelating agents have been considered as promising tools in food technology to protect food from chemical, enzymatic, and oxidative reactions. However, their use in food fortification is still very limited. A few studies have explored the potential usefulness of chelating Zn with biological agents (amino acid, peptide, and solubilizing bacteria) in the biofortification of staple foods such as cereals and cereal-based products. These studies have shown that biofortification of staple foods with Zn in the chelated form has lower phytotoxicity than conventional biofortification [76]. It also improves Zn content and enhances the growth, productivity, and nutritional quality of the plant-based products [77,78]. The formation of chelate complexes of metals with biological structures makes it possible to obtain highly stable forms of trace elements. Polysaccharides and peptides derived from plants are considered the most recent and interesting biological materials. Used as a Zn chelator, they offer improved Zn absorption, bioavailability, and bioactivity without toxicity or hazards (Table 1).

- Zinc-chelating polysaccharides

The natural polysaccharide–Zn complex has significant advantages as a biological Zn supplement. Recent investigations have explored the effective Zn chelating capacity of many polysaccharide types extracted from different plant and mushroom species such as Zingiber officinale roscoe and Dictyophora indusiata. In vivo and in vitro experiments have shown that natural polysaccharide-chelating Zn complexes were more efficient in terms of Zn absorption and bioavailability and demonstrated better bioactivity than inorganic and organic Zn supplements. In fact, polysaccharide–Zn complexes were reported to have significant antioxidant activities, anti-proliferative activity, and anti-diabetic effects. They modulate immunity and can be considered as potential candidates to treat Zn deficiency (Table 1).

- Zinc-chelating peptides

According to several recent studies, some natural peptides have the ability to bind to Zn and improve its absorption and bioavailability by maintaining it in a soluble form in basic intestinal conditions. Zn-chelating peptide complexes have therefore been considered as an effective functional ingredient to enhance dietary Zn bioavailability [79,80]. These recent studies have demonstrated that food fortification with Zn–peptide complexes increases the solubility and absorption of Zn in the intestinal tract and thereby improves its bioavailability and bioactivity in comparison with conventional Zn supplementation methods (Table 1). Peptide chelators have been identified and extracted from different animal and vegetable sources including milk, wheat germ, and oysters (Table 1). Wang et al. [81] showed that yak milk casein hydrolysate (YCH) has the ability to bind efficiently with Zn ions and produce a complex making Zn more soluble than Zn acetate under simulated intestinal conditions. Similarly, the studies of [82] investigated the mechanism by which casein phosphopeptides (CPPs) increase Zn bioavailability both in vitro and in vivo. The results showed that Zn absorption from the CPP–Zn complex was much higher than from ZnSO_4_ for Dawley rat pups, possibly because the Zn bound to CPPs was protected from insolubilization during digestion or interactions with other food in the gut.

Recently, Zn-chelating peptides extracted from oysters have attracted wide attention [64,83]. Oysters are considered one of the richest food sources for Zn. One possible Zn binding peptide (HLRQEEKEEVTVGSLK) has already been identified from oyster protein hydrolysates. The characterization of the complex, with regard to the Zn solubility and the mechanism of Zn absorption in vitro using Caco-2 cells, has also been investigated [64,84]. All these studies support the potential application of a peptide–Zn complex derived from oysters to enhance Zn bioavailability in the presence of phytic acid.

**Table 1:** Recently reported zinc-chelating biological agents and their effect on zinc bioavailability and bioactivity.

Zn Chelator Type	Zn Chelator Biological Source/Zn Precursors Used	Effect of the Zn Chelating Complex on Zn Bioavailability and Bioactivity	References
Polysaccharide (Ps)	Ginger (*Zingiber officinale* Roscoe) peel powder; pumpkin skin (*Cucurbita moschata*)/ZnSO_4_	Ps–Zn complex could be used as a safe and effective form of Zn supplementation to prevent inflammatory reaction induced by copper sulfate (CuSO_4_) in zebrafish.	[85]
	Prepared *Athelia rolfsii*/Zn^2+^	In vivo experiments in mice showed that Ps–Zn complexes were more effective than inorganic and organic Zn supplements in treating Zn deficiency and improving antioxidant activities.	[86]
	*Dictyophora indusiata*; *Prunella vulgaris*/Zinc Chloride (ZnCl_2_); Zinc acetate (Zn(CH_3_CO_2_)_2_)	Ps–Zn complexes were reported to have significant anti-proliferative activity against a group of human cancer cell lines via several biological pathways.	[87,88]
	Fresh garlic/ZnSO_4_	Ps–Zn complex could be considered as a potential form of Zn supplementation to alleviate the toxic effect induced by Zn deficiency in mice, including oxidative stress.	[89]
Peptides (Pp)	Oyster/ZnSO_4_	Pp-Zn complex could significantly enhance Zn bioavailability in vitro on Caco-2 cells and enhance Zn solubility during simulated gastrointestinal digestion in comparison to the commonly used ZnSO_4_.	[64,84]
	Sea cucumber (*stichopus ja ponicus*); Scallop adductor (*Patinopecten yessoensis*)/ZnSO_4_	In vitro experiments on Caco-2 cells suggest that marine-animal-derived peptides could be considered as a potential and safe Zn-chelating agents to enhance Zn absorption and bioavailability.	[90,91]
	Wheat/ZnSO_4_	In vitro experiments on Caco-2 cells suggest that Zn-chelating peptides from wheat germ protein hydrolysates possessed higher Zn bioavailability than ZnSO_4_.	[92]

### 6.2. Micro- and Nanoencapsulation of Zinc

The use of nano- or microcapsules is a novel strategy used in agriculture and the food industry for food fortification/biofortification and for the packaging and coating of food products.

- Zinc microencapsulation

Microencapsulation is a modern technology that has been used to encapsulate and mask the metallic taste of minerals [93]. Many methods have been described and used for the encapsulation of minerals such Zn [94,95]. Spray drying is the most common approach for mineral microencapsulation due to its low cost and high production rate. Spray drying has a number of advantages in using a diverse range of covering materials for encapsulation. These advantages have established spray drying as one of the few unit operations that can be easily scalable and employed on industrial scales [95]. Recently, spray-dried Zn compound microencapsulation has been used in food fortification [95,96].

- Zinc nanoencapsulation

Nanotechnology is a modern technology that deals with materials (atoms and molecules) at sizes of 1–100 nm. The use of Zn at the nano scale has recently been applied in the food industry including food packaging and processing [97]. Several chemical and physical approaches have been developed to synthetize Zn nanoparticles (Zn NPs) including precipitations, vapor deposition, laser ablation, and hydrothermal processes [98,99]. However, these methods present a safety risk to the environment as well as to human health. To alleviate Zn NP toxicity, a green synthesis process has been developed [100,101]. The green technology uses natural materials extracted from plants and bacteria to create nanoparticles [102]. In addition to being environmentally friendly, this process has a lower cost than traditional chemical and physical methods [103]. Many recent in vitro studies have described the potential anti-microbial, anti-oxidant, anti-inflammatory, and anti-cancer properties of Zn NPs as reviewed by [99], suggesting their effective role in enhancing and improving the bioaccessibility of Zn to the cells. The biofortification of animal feedstuffs and plants, as well as foliar/soil fortification with Zn NPs, has recently been described. These studies have demonstrated that biofortification with Zn NPs ameliorates the nutritional quality of animal products (meat, milk, eggs, etc.) [104] and improves the growth and productivity of cereal-based crops. A few studies have investigated the direct supplementation of Zn NPs in dairy and meat/fish-based products (Table 2).

Overall, it is critically important to understand the structure–function relationship of zinc-chelating peptides or zinc encapsulated forms in food fortification processes. Their behavior during gastrointestinal digestion after intake of fortified food is also critically relevant concerning human nutrition and health promotion as reviewed by [80,119]. Encapsulation involves the formation of a protective barrier around the Zn, which helps to prevent interactions with other food components that could reduce its bioavailability [119]. Chelation involves the formation of stable complexes between Zn and other molecules that can enhance the solubility and stability of Zn in the food matrix [80]. As the effectiveness of encapsulation and chelation can vary depending on the food matrix and the method of fortification used, further research is needed to identify the most effective methods for improving Zn bioavailability in fortified foods and to develop strategies to improve Zn nutrition.

## 7. Conclusions

Zn is recognized as a physiological metal essential to the metabolism of most living beings and plays a multifaceted role in biological processes. Zn is considered as a potent immunomodulatory element and its deficiency in the body is linked with the alteration of immunological functions and viral infections including SARS-COV-2, which is responsible for COVID-19. Recent research confirms that optimal intake, or appropriate supplementation of Zn, should be considered as part of the strategy to reduce COVID-19 effects. Controlling the amount of this element in a diet is especially important for populations at risk of zinc deficiency such as infants and young children, women during pregnancy, and the elderly. Various food technology approaches are used to compensate for Zn deficiencies. Promising trends in food fortification/biofortification with Zn include: the formation of chelate complexes of metals with biological structures extracted from plants (peptides and polysaccharides), creating highly stable forms of trace elements, the encapsulation of mineral elements in microcapsules that protect against interaction with the food system and prevent the destruction of the component in the digestive tract, and the use of food additives in the form of nanoparticles that combine high bioavailability due to particle size with excellent sensory characteristics. These recent food fortification/biofortification approaches with Zn could be promising tools to enhance Zn bioavailability in the human body and therefore prevent Zn deficiency. Furthermore, given the significant immunomodulatory effect of Zn in the body, improving Zn bioavailability will be advantageous for maintaining and boosting the immune system health, which will result in, amongst other things, preventing viral infections such as those involved in COVID-19.

## Figures and Tables

**Table 2:** Recent reports about the use of Zn NPs in food fortification/biofortification.

Food Product Fortified/Biofortified with Zn NPs	Fortification/Biofortification Methods	Consequence of Food Fortification/Biofortification with Zn NPs	References
Cereal-based products (rice, wheat, etc.)	Biofortification (Foliar fortification)	Foliar application of Zn NPs (synthetized by green technology) at low and moderate doses enhanced the growth, yield, and the quality of some cereal crops (rice, wheat, and amaranth).	[105,106,107]
Dairy-based products (milk, yoghurt, etc.)	Fortification	Fortification of dairy products with Zn NPs showed advantages over conventional processes in terms of microbial profile, physicochemical, and rheological properties right after manufacturing and during refrigerated storage. In vitro digestion analysis of the dairy product fortified with Zn NPs showed more solubility than conventional fortification.	[21,108,109]
	Biofortification	The administration of Zn NPs in cattle feed reduces the number of somatic cells in milk, increases Zn concentration, and improves milk production in comparison with conventional Zn supplementation.	[104,110,111]
Meat- and fish-based products	Fortification	The uses of Zn NPs (synthetized by green technology) enhance shrimps’ biopreservation during refrigerated storage by improving sensorial qualities and decreasing the microbial profile of the animal product.	[112]
	Biofortification	Results indicated that feeding animals (rabbits, pigs, etc.) with Zn NPs increases Zn absorption and bioavailability in comparison to regular Zn sources and has a positive effect on production efficiency, quality, and characteristics (physicochemical properties, antioxidant status, Zn content, etc.) of eggs, meat, and bones of feeding animals.	[113,114,115,116,117,118]

## Data Availability

Not applicable.
